# Acceptability of a Mobile Phone–Based Augmented Reality Game for Rehabilitation of Patients With Upper Limb Deficits from Stroke: Case Study

**DOI:** 10.2196/17822

**Published:** 2020-09-02

**Authors:** Nina LaPiana, Alvin Duong, Alex Lee, Leon Alschitz, Rafael M L Silva, Jody Early, Aaron Bunnell, Pierre Mourad

**Affiliations:** 1 Department of Neurological Surgery University of Washington Seattle, WA United States; 2 Nursing and Health Studies University of Washington Bothell Bothell, WA United States; 3 Department of Engineering and Mathematics University of Washington Bothell Bothell, WA United States; 4 Rehabilitation Medicine Clinic Harborview Medical Center Seattle, WA United States

**Keywords:** augmented reality, stroke, upper limb rehabilitation, gamification, motor rehabilitation, motivation, participation

## Abstract

**Background:**

Upper limb functional deficits are common after stroke and result from motor weakness, ataxia, spasticity, spatial neglect, and poor stamina. Past studies employing a range of commercial gaming systems to deliver rehabilitation to stroke patients provided short-term efficacy but have not yet demonstrated whether or not those games are acceptable, that is, motivational, comfortable, and engaging, which are all necessary for potential adoption and use by patients.

**Objective:**

The goal of the study was to assess the acceptability of a smartphone-based augmented reality game as a means of delivering stroke rehabilitation for patients with upper limb motor function loss.

**Methods:**

Patients aged 50 to 70 years, all of whom experienced motor deficits after acute ischemic stroke, participated in 3 optional therapy sessions using augmented reality therapeutic gaming over the course of 1 week, targeting deficits in upper extremity strength and range of motion. After completion of the game, we administered a 16-item questionnaire to the patients to assess the game’s acceptability; 8 questions were answered by rating on a scale from 1 (very negative experience) to 5 (very positive experience); 8 questions were qualitative.

**Results:**

Patients (n=5) completed a total of 23 out of 45 scheduled augmented reality game sessions, with patient fatigue as the primary factor for uncompleted sessions. Each patient consented to 9 potential game sessions and completed a mean of 4.6 (SE 1.3) games. Of the 5 patients, 4 (80%) completed the questionnaire at the end of their final gaming session. Of note, patients were motivated to continue to the end of a given gaming session (mean 4.25, 95% CI 3.31-5.19), to try other game-based therapies (mean 3.75, 95% CI 2.81-4.69), to do another session (mean 3.50, 95% CI 2.93-4.07), and to perform other daily rehabilitation exercises (mean 3.25, 95% CI 2.76-3.74). In addition, participants gave mean scores of 4.00 (95% CI 2.87-5.13) for overall experience; 4.25 (95% CI 3.31-5.19) for comfort; 3.25 (95% CI 2.31-4.19) for finding the study fun, enjoyable, and engaging; and 3.50 (95% CI 2.52-4.48) for believing the technology could help them reach their rehabilitation goals. For each of the 4 patients, their reported scores were statistically significantly higher than those generated by a random sampling of values (patient 1: *P*=.04; patient 2: *P*=.04; patient 4: *P*=.004; patient 5: *P*=.04).

**Conclusions:**

Based on the questionnaire scores, the patients with upper limb motor deficits following stroke who participated in our case study found our augmented reality game motivating, comfortable, engaging, and tolerable. Improvements in augmented reality technology motivated by this case study may one day allow patients to work with improved versions of this therapy independently in their own home. We therefore anticipate that smartphone-based augmented reality gaming systems may eventually provide useful postdischarge self-treatment as a supplement to professional therapy for patients with upper limb deficiencies from stroke.

## Introduction

### Background

Stroke induces a variety of functional impairments, as well as pain and other ailments, depending on its type and location [1]. Common deficits associated with ischemic stroke include motor function, spatial neglect, and psychological changes [1]. Motor function deficits after stroke often include partial or total loss of function of the upper or lower limbs on a given side, with associated muscle weakness, poor stamina, lack of muscle control, and even paralysis [2]. These deficits impact the patient’s independent lifestyle and decrease their performance of activities of daily living [1]. According to the National Institute of Neurological Disorders and Stroke, the most important part of rehabilitation programs is “carefully directed, well-focused, repetitive practice [3].”

### Prior Work

Patients who engage in rigorous, time-intensive, and challenging therapeutic exercises after ischemic stroke tend to experience greater functional recovery, while if ignored or insufficiently treated, impairments may remain [4,5]. The dosage of motor skill practice correlates to the extent of motor recovery following a stroke [4]. In addition, the type of therapy delivered relative to patient’s impairment determines outcomes after therapy. For example, for those who have upper limb motor impairment, best therapeutic practice modifies the prescribed exercises as the patient’s symptoms evolve [5,6]. Regrettably, patients report their experiences of conventional repetitive stroke rehabilitation therapies as tedious and difficult to hold their interest, which conflicts with the fact that patient motivation is often required to obtain good clinical outcomes [7-10].

Rehabilitation doctors and medical staff, therefore, face a significant problem: how can they provide high intensity therapy in large quantities for upper limb impairments with this seemingly intrinsic motivational deficit? Especially problematic are patient’s therapeutic needs after their discharge from the hospital—their therapeutic needs still exist, but medical staff have substantially reduced access to the patient to provide targeted care. Given the difficulty of this problem, an insufficient percentage of patients regain the full functional potential of their upper limb after ischemic stroke [11]. This regrettable outcome motivates an ongoing search for new therapeutic approaches that provide acceptable (motivational, comfortable, and engaging) experiences, hence, effective therapy, especially at the patient’s home. 

Use of commercial augmented reality devices has found recent application in stroke rehabilitation using existing expensive commercial headsets [4,6-17]. However, there are few studies that assay the acceptability of augmented reality gaming system–based patient rehabilitation after stroke [10,12,17-19], and then, only in a cursory fashion. For example, 30 patients recovering from stroke were surveyed for their opinions on game-based rehabilitation, and the researchers concluded that though games for patients recovering from stroke existed, they were primarily designed for efficacy, not entertainment [10]; they suggest investing in a single, affordable gaming platform for patient rehabilitation after stroke that also focuses on entertainment and provides diverse gaming content [10]. Augmented reality technology and an upper-limb assistive device were tested on 3 individuals recovering from stroke for 6 weeks, and the study reported that both the user and therapist believed that their augmented reality environment was user friendly due to the lightness of the assistive devices and the simplicity of set-up [18]. Finally, a study of 4 patients recovering from stroke who were exposed to several gaming platforms reported that manually adjusting the difficulty of games to provide a challenge and creating games with deeper story lines helped the patients stay motivated to perform their gaming exercises [17]. To the best of our knowledge, our case study is the first of its kind that analyzes the opinions of patients recovering from stroke regarding the problems of current augmented reality–specific game-based rehabilitation systems to provides insight into future designs of augmented reality game-based stroke rehabilitation systems. Augmented reality, provided by one of a variety of device designs, represents one such approach. Augmented reality projects a live camera view of a user’s environment and computer-generated objects with a variety of properties—movement and sound, typically. As an example, Pokémon Go, a smartphone-based augmented reality game, has had documented success sustaining the interest of users for extended periods of time while consistently increasing their physical activity [13], making augmented reality a prime candidate for facilitating otherwise tedious therapy.

### Hypothesis

Since patient motivation often drives a larger dosage of rehabilitation therapy, hence, improved clinical outcomes [20,21], we hypothesized that augmented reality deployed on a relatively inexpensive and readily available platform—a smartphone—could provide a motivational, comfortable, and engaging rehabilitation experience. To test this hypothesis, we first developed a candidate rehabilitation game on a smartphone that could encourage a patient’s hand motions through use of simple visual cues with a custom-made app. We then asked patients with acute upper-motor stroke to use this system and report their experiences via a questionnaire that assayed the acceptability of the game in terms of motivation to continue to play, comfort, and engagement.

## Methods

### Overview

This acceptability study was conducted at Harborview Medical Center in Seattle, Washington from November 2018 to March 2019. Inpatients who were recovering from an acute ischemic stroke participated and provided consent. These patients had impaired strength as determined by physical and occupational therapists. To be included in the study, they had to have at least antigravity strength in deltoid or biceps muscles as well as the ability to perform internal and external shoulder rotations. All patients in this study had a Medical Research Council manual muscle score of 3 or 4 in the affected limb.

### Intervention

**Figure 1:**
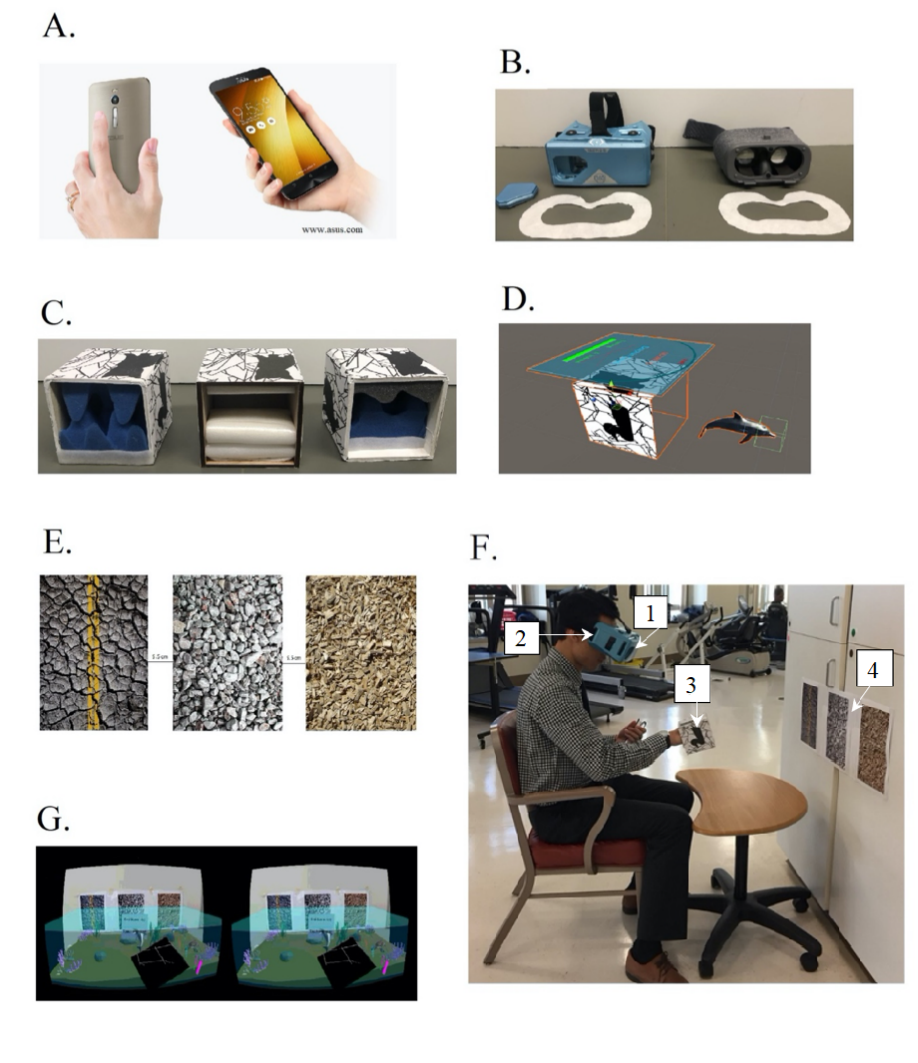
(A) phone: Asus Zenfone 2, phone operating system: Android 7 Nougat, Unity version: 2018.2.10, developer operating system: Windows 10; (B) headsets: Google Daydream (left) Merge augmented reality/virtual reality goggles (right); (C) controllers with various grip sizes consisting of soft foam inserts; (D) virtual dolphin avatar; (E) image target; (F) study staff during game play with (1) smartphone (2) headset (3) controller (4) image target; (G) user experience.

We designed and built an augmented reality game using Unity (Unity Technologies) that is deployable on any modern smartphone with a camera (Table 1 and Figure 1). The game presents users with a view of an augmented reality dolphin swimming under the ocean with the task of capturing fish and feeding turtles, worn on the hand associated with the upper-limb deficit (Multimedia Appendix 1). To experience the game, patients wore an augmented reality headset, which did not obscure the camera mounted on the phone, and a custom device on their hand. We used two headsets—the Google Daydream headset, which required us to remove the front panel that held the phone in place, and the Merge augmented reality/virtual reality headset, which did not require any modification (Figure 1). The game also required users to place the hand associated with their motor deficits within a padded box that replaced their hand as seen in augmented reality with a dolphin (Figure 1). Finally, we required the user to look at a complex landscape through their headset while wearing the padded box and while playing the game. Instead of holding the phone, the headset supported the phone for the user. We built customized controllers with different interior sizes that changed the effective grip strength of the controller; this was important because our patients’ ability to hold the controllers varied. Viewing the complex landscape through the augmented reality system caused our software to create a seascape that contained a turtle, fish, and other underwater flora and fauna (Multimedia Appendix 1). Successful placement of the dolphin over a fish allowed the dolphin to capture the fish. Placement of the dolphin plus fish over the turtle allowed the user to feed the turtle, thereby winning points.

Notably, we used the TeamViewer (TeamViewer AG) app to project the screen view of the patient from the phone to a laptop, so we could see the patient’s view with, however, the complex landscape was also projected in the background, so we could check the viewer’s alignment with the landscape while they played (Figure 1).

Set-up of the game, to ensure that system function was verified, occurred prior to patients using the system. Patients followed verbal directions and instructions from study staff on how to use the system, facilitated by demonstration of the game using the TeamViewer app. Examples of directions included how to start the game, the actions required to pick up the fish, and how to colocate the dolphin plus fish with the turtle for point accumulation. Some patients required physical assistance to adjust the view of the environment. Examples of physical assistance included moving the patient’s chair or wheelchair closer or farther away from the images recognized by the camera (Figure 1).

**Table 1:** Vuforia compatible mobile devices.

Device operating system			Development operating system			Unity version	
Android	4.4+	Windows		7+	Windows		2018.2+
iOS	9+	OS X		10.13+	OS X		2018.2+

### Procedures

In this study, we asked the patients to complete 3 30-minute multigaming sessions on separate days over the course of 1 week while the patients continued their standard therapy schedule. Sessions were conducted after all prescribed therapy so as not to interfere with the patient’s schedule. We gave patients the choice to start with their affected or unaffected hand each game while encouraging them to try their affected hand.

Before gaming, we fitted the patient with a nitrile glove. Before and after every gaming session the headsets, smartphone, and TeamViewer laptop were wiped with hospital-grade bleach wipes and left to dry for at least 2 minutes. Each game lasted approximately 4 minutes and was always played in a seated position. The game displayed a score to the user in real time and at the end of the game. We recorded final scores for each user and game. We also recorded which hand the patient used for each game. After each game, we removed the headset and restarted the game on the mobile phone. During a 2-minute break after each game, we asked the user to describe their experience and took notes on their verbal commentary. Our goal was for each patient to play 9 games over the course of 1 week.

### Outcome Measures

To test our hypothesis, we provided an 8-question questionnaire that assayed the patient’s perceived engagement and the acceptability of their experience at the end of the third completed session. This questionnaire assessed the patient’s feelings regarding the gaming experience, their perceived acceptability of the experience in terms of its motivational qualities, their perception of comfortability, and their enjoyment of the game (1 - very negative, 2 - negative, 3 - neutral, 4 - positive, 5 - very positive). We also asked an additional 8 questions about their previous experiences with videogames and the likelihood that such a system would be used by them in the future.

We determined the statistical significance of the answers to the first 8 questions by comparing the numerical distribution of each patient’s answers against those of a random distribution of answers to the same questions (Multimedia Appendix 2).

## Results

### Participants

We recruited 5 patients in rehabilitation into the study. Patient 1 was a 58-year-old man with right thalamic intraparenchymal hemorrhage and presented with left-sided hemiparesis with major fatigue in the left arm post–daily therapy. Patient 2 was a 70-year-old man with bilateral right>left pontine ischemic stroke with visual impairment and double vision in the left eye. The patient had limited range of motion in his right arm and hand with function that increased sufficiently after admittance that he met our inclusion criteria. Patient 3 was a 67-year-old man with left middle cerebral artery ischemic stroke. His left arm was his nondominant hand and was self-reported to be functioning at “100%.” His affected right hand was his dominant hand and self-reported to be functioning “at 50%.” Patient 4 was a 50-year-old woman with right thalamic intraparenchymal hemorrhage. The patient’s left hand was affected. Patient 5 was a 65-year-old man with right anterior cerebral artery ischemic stroke. The patient typically wore glasses. He said it was “small double vision/blurriness in his right eye.” 

The mean age of the 5 patients was 62 years old. Of the 4 patients who completed our questionnaire, all 4 lacked experience with augmented reality while 3 out of 4 had no experience with videogames. Finally, 1 patient reported little experience with videogames (less than 3 times in 24 months).

### Intervention

Table 2 summarizes patient participation. All patients completed at least one game session with their affected hand. Together, patients completed 23 out of the 45 game sessions. Each game module lasted approximately 4 minutes. Each patient had consented to 9 possible game sessions. The mean number of games played by each patient was 4.6 (SE 1.3).

**Table 2:** Patient game sessions with the number of points scored, the hand chosen by the patient for play during a given game, and the functional status of the hand.

Patient games		Session 1		Session 2		Session 3	
		Score	Hand	Score	Hand	Score	Hand
**Patient 1**							
	Game 1	260	right (unaffected)	380	both	110	both
	Game 2	210	both	0	both	240	both
	Game 3	—	fatigue	—	N/A^a^	320	both
**Patient 2**							
	Game 1	0	right (affected)	0	right (affected)	0	right (affected)
	Game 2	—	N/A	—	N/A	—	N/A
	Game 3	—	N/A	—	N/A	—	N/A
**Patient 3^b^**							
	Game 1	0	right (affected)	—	N/A	—	N/A
	Game 2	—	fatigue	—	N/A	—	N/A
	Game 3	—	fatigue	—	N/A	—	N/A
**Patient 4**							
	Game 1	0	right (unaffected)	190	right (unaffected)	0	right (unaffected)
	Game 2	0	left (affected)	150	right (unaffected)	120	right (unaffected)
	Game 3	90	right (unaffected)	—	malfunction	30	right (unaffected)
**Patient 5**							
	Game 1	210	left (affected)	130	left (affected)	—	N/A
	Game 2	280	right (unaffected)	450	right (unaffected)	—	N/A
	Game 3	—	N/A	—	N/A	—	N/A

^a^N/A: not applicable because the patient dropped out.

^b^Patient was discharged early.

### Adverse Events

No adverse medical events occurred during our study. Patient 2, despite the relative severity of his reduced function, felt sufficiently motivated to try the game for each of the 3 sessions. Patient 3 was discharged early, and therefore, did not complete 6 of 45 possible sessions (13%) across all patients. A total of 3 of 45 sessions (7%) from 2 patients were incomplete due to their fatigue from daily rehabilitation sessions; 12 of 45 sessions (27%) from 3 patients were incomplete due to discontinuation of the study session. Finally, 1 of 45 sessions (2%) was incomplete due to malfunction of the gaming apparatus.

### Patient Satisfaction—Quantitative Results

**Figure 2:**
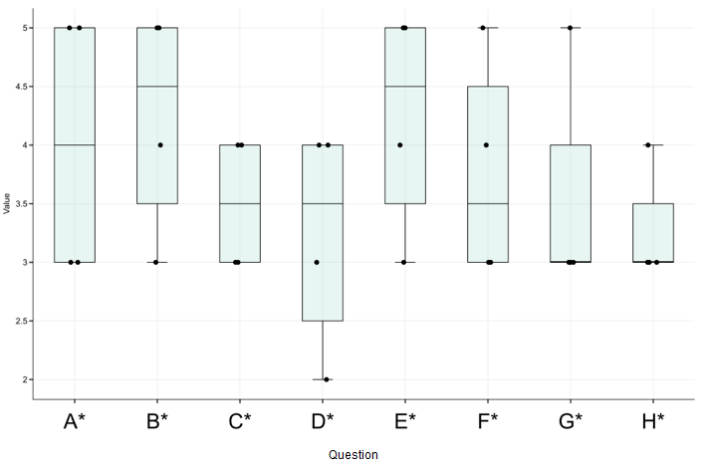
Patient ratings on a scale from 1 (very negative) to 5 (very positive).

Patients 1, 2, 4, and 5 completed the questionnaire that we gave at the end of their final session; patient 3 was discharged before completion of their participation in the study. Table 3 shows the individual scores while Figure 2 shows the distribution of the scores. Organized by theme, the patients reported a mean score of 4.25 (95% CI 3.31-5.19) for motivation to follow the instructions and finish the augmented reality experience to the end of a given gaming session, 3.75 (95% CI 2.81-4.69) for motivation to try other game-based therapies, 3.50 (95% CI 2.93-4.07) for desire to do another session, and 3.25 (95% CI 2.76-3.74) for motivation to perform other exercises in support of their daily rehabilitation. Organized by comfort, the patients reported an average score of 4.00 (95% CI 2.87-5.13) for the overall experience, 4.25 (95% CI 3.31-5.19) for comfort. Organized by engagement, patients reported an average score of 3.25 (95% CI 2.31-4.19) for finding the study fun, enjoyable, and engaging; and 3.50 (95% CI 2.52-4.48) for believing this technology could help them reach their rehabilitation goals.

*P* values for each patient are reported in Table 4. For each of the 4 patients, the reported scores were statistically significantly higher than those generated by a random sampling of values (patient 1: *P*=.04; patient 2: *P*=.04; patient 4: *P*=.004; patient 5: *P=*.04) consistent with the interpretation that the patients found our augmented reality game acceptable.

**Table 3:** Results and response comparison of acceptability questionnaire.

Assessment		Patient				Score	
		1	2	3	4		Mean (95% CI)
**Questions**							
	A. How do you feel about the experience you just performed?	5	3	5	3		4.00 (2.87, 5.13)
	B. How would you rate the comfort of the experience?	4	3	5	5		4.25 (3.31, 5.19)
	C. How do you feel about doing another session?	4	3	4	3		3.50 (2.93, 4.07)
	D. The game was fun, enjoyable and/or engaging	4	2	4	3		3.25 (2.31, 4.19)
	E. During the session, I was motivated to follow the instructions and keep playing the game until the end	5	3	5	4		4.25 (3.31, 5.19)
	F. This session made me feel motivated to try other game-based therapies	3	3	5	4		3.75 (2.81, 4.69)
	G. I think gaming with this technology will help me reach my rehabilitation goals	3	3	5	3		3.50 (2.52, 4.48)
	H. The game also made me feel motivated to perform other exercises in support of my rehabilitation	3	3	3	4		3.25 (2.76, 3.74)
*P* value (comparison with random sampling)		.04	.04	.004	.04		

**Table 4:** Patient comments.

Question	Response
Did you experience any negative symptoms during or after game play such as nausea, dizziness, pain, fatigue, headache, general discomfort, etc?	“no” [Patient 1] “no” [Patient 2] “no” [Patient 4] “none” [Patient 5]
Did you have trouble playing the game? If yes, please describe.	“sometimes it was difficult to reach the far turtle to drop off fish.” [Patient 1] “yes” [Patient 2] “was difficult to navigate the dolphin to the target” [Patient 4] “only in lining up the controller to the camera” [Patient 5]
Please provide any additional comments which will help us understand your experience while using the Augmented Reality system.	“better controllers to be able to hit the targets maybe a glove of some kind. also in the beginning to help stroke patients maybe simplify not have moving targets something of a push the target on the first level then move to upper levels of moving targets as thing progress for the patients.” [Patient 4] “Hard for me to judge im 65 no experience” [Patient 5]

### Patient Questionnaire Comments

Here we report all of the patient comments offered to us. A common theme expressed by the patients was their desire to have better hand-held controllers for the game than the custom boxes, since the boxes made it difficult to navigate the dolphin to the target. Patient 3 did not complete the questionnaire due to early discharge. Some patients chose not to comment on all 3 open response questions.

## Discussion

### Principal Results

In our observational case study on the acceptability of a therapeutic smartphone-based augmented reality gaming for patients with upper limb motor impairment from acute ischemic stroke, 5 users with ages in the range of 50 to 70 years volunteered and completed a total of 23 of 45 possible gaming sessions; 4 patients who remained in the rehabilitation unit completed a questionnaire after completing their gaming sessions and before their discharge. The patients rated their motivation, comfort, the value of their experience, and desire to play another round of the game the highest. In contrast, the lowest rated aspects were enjoyability of the game itself and motivation to try other rehabilitation technologies. It is worth noting, however, that all scores were equal to or better than neutral scores. In this study, point accumulation was unrelated to acceptability. Nonetheless, even when a patient did not earn any points during a given game, they reported that our augmented reality game was generally comfortable and that they were motivated to try to play the game again.

### Limitations

While 3 out of 4 of the patients engaged in all 3 of their gaming sessions, 1 patient left the study due to their early discharge, which reduced the total number of games by 13%. Enrolling more patients would address this issue.

Another limitation was the quality of the game, affected in part by limitations imposed by our use of state-of-the-art but limited camera control software and our box-shaped controller, whose size—necessitated by the need to encompass the hand of the patient—made it difficult for users to select a moving target with their affected hand even with additional verbal cues. Compared to virtual reality, state-of-the-art augmented reality programs deployable on smartphones currently lack features and stability mainly due to outdated hardware specifications. This resulted in some frustration and lower participant satisfaction scores as reflected by questionnaire responses.

Given our focus on assaying the patient’s experience of the augmented reality game, another limitation was that we did not include a comparison of the patient’s experience with standard therapy plus the game versus a separate group of patients who experienced only their standard therapy. Future work will include this comparison as part of an efficacy study, once we improve the mechanics of the game itself.

The fatigue experienced by patients during the day of their sessions also impacted their experience with our technology. Recall that all test patients experienced our augmented reality technology after completing their regularly schedule therapy. While all patients reported fatigue, 2 out of 5 patients dropped out of at least 1 game session due to postrehabilitation fatigue. 

Also, most of our patients who participated in the study had little to no experience with augmented reality and videogames. They, therefore, could not compare our game with other such games. This minimal experience with augmented and virtual reality is typical of this demographic of adults 50 years and older [22], a fact that game designs must take into account when considering therapeutic applications. They can do so by developing a greater understanding of what can motivate a patient to do more sessions and by establishing a closer alignment of game movements with their rehabilitation goals.

### Future Studies

With this case study, we report our initial findings regarding the acceptability of our augmented reality approach to acute stroke rehabilitation, in anticipation of future studies that would test for efficacy. This allows us to gauge the requirements for more formal, and eventually, long-term studies of augmented reality game rehabilitation in an older stroke population. The next logical step is to refine the augmented reality platform and therapeutic gaming software to make it more engaging and with more robust functionality. For example, body ownership studies suggest that the visual feedback a patient receives from viewing their own limb may be more beneficial while recovering from stroke than that of an unrelated virtual component [16,23,24]. We will therefore explore reduction of the hand marker so the hand itself has more visual impact, perhaps by using the patient’s hand instead of an avatar during the gaming experience. Other improvements that we would like to make when we repeat this study are to include increasing difficulty as the user improves their range of motion and speed (beginning with stationary targets), multiple environments and levels for the user, and more visually effective controllers. We may also incorporate optional bilateral gaming elements. In our next therapeutic gaming technology, range of motion measurements will be implemented into our hardware and compared with patient Wolf Motor Function [25] or Fugl-Meyer [26] range of motion data. With such improvements in hand, a prospective efficacy comparison of standard therapy to augmented reality therapy with standard therapy and home-setting studies are anticipated.

### Conclusion

Members of an older population recovering from acute stroke found smartphone-based augmented reality game targeting therapy of the upper limb acceptable. We also identified improvements to the experience that will inform the next study of this potential therapy. Of importance, such a gaming set-up could be used for home-based therapy due to its relatively low cost and ease of use. Therefore, this work informs the next formal study of this technology for upper limb motor rehabilitation. We anticipate eventual study of this technology in the home setting, after acute rehabilitation, which is of interest since patients spend most of their time at home performing rehabilitation exercises after a stroke. We anticipate that a sufficiently engaging smartphone-based game will lead to more use and greater therapeutic benefit experienced by the patient, as well as possibly improved clinical outcomes for patients.

## Appendix

Multimedia Appendix 1 User experience video.

Multimedia Appendix 2 Patient feedback RedCap questionnaire.
