# Supplementary material for: Acceptability of a Mobile Phone–Based Augmented Reality Game for Rehabilitation of Patients With Upper Limb Deficits from Stroke: Case Study
Source: JMIR Rehabil Assist Technol. 2020 Sep 2;7(2):e17822. doi: 10.2196/17822 (PMC7495251; doi:10.2196/17822)
Supplement: Multimedia Appendix 2 [file rehab_v7i2e17822_app2.pdf]

Acceptability Questionnaire for Patients
Augmented Reality

1. What is your highest level of formal education?

_No Formal Education
_High School or equivalent
_Some College
_Advanced Degree (Associate, Bachelor, Master, PhD,…)

2. If you have formal education, did you take classes on development or advanced use of technology? For example, if you were trained on computer programming, electronics design, 3D modeling, or similar tasks.

_Yes
_No

3. Have you ever tried Augmented Reality?

_Yes
_No

4. How much experience with Augmented Reality do you have?

_Unexperienced (It has been a long time, or I have never used it prior to this experiment)
_Little experience (I have tried it less than 3 times in the past 24 months)
_Experienced (I have used it a substantial number of times within the past 24 months)
_Expert (I have developed and/or evaluated Augmented Reality applications before)

5. What is your experience with Video Games?

_Limited (I don't remember the last time I played any video game.)
_Little experience (I have played video games less than 3 times in the past 24 months)
_Experienced (I play video games regularly, or have played it in several occasions in the past 24 months)
_Expert (I work on developing or evaluating video games)

Describing the Experience

6. How do you feel about the experience you just performed?

5 Very Positive
4 Positive
3 Neutral
2 Negative
1 Very Negative

7. How would you rate the comfort of the experience?

5 Very Positive
4 Positive
3 Neutral
2 Negative
1 Very Negative

8. Did you experience any negative symptoms during or after game play such as nausea, dizziness, pain, fatigue, headache, general discomfort, etc.?

_No
_Yes. Please briefly describe:

____

9. Did you have trouble playing the game?

_No
_Yes. Please briefly describe:

____

Looking forward

10. How do you feel about doing another session?

5 Very Positive ("I would do another session right now")
4 Positive ("I would be willing to participate in another session in the future")
3 Neutral ("I don't have any particular feelings about participating in a future session")
2 Negative ("I probably would not participate in future similar sessions")
1 Very Negative ("I would not be willing to participate in any future sessions")

<u>Subjective commentary</u>
<u>Please, indicate how much you agree with the following statements</u>

11. The game was fun, enjoyable and/or engaging.

5 Strongly agree
4 Agree
3 Neither agree nor disagree
2 Disagree
1 Strongly Disagree

12. During the session, I was motivated to follow the instructions and keep playing the game until the end.

5 Strongly agree
4 Agree
3 Neither agree nor disagree
2 Disagree
1 Strongly Disagree

13. This session made me feel motivated to try other game-based therapies.

5 Strongly agree
4 Agree
3 Neither agree nor disagree
2 Disagree
1 Strongly Disagree

14. I think gaming with this technology will help me reach my rehabilitation goals.

5 Strongly agree
4 Agree
3 Neither agree nor disagree
2 Disagree
1 Strongly Disagree

15. The game also made me feel motivated to perform other exercises in support of my rehabilitation.

5 Strongly agree
4 Agree
3 Neither agree nor disagree
2 Disagree
1 Strongly Disagree

16. Please provide any additional comments which will help us understand your experience while using the Augmented Reality system:

____
